# Eye Movement Desensitization and Reprocessing Integrative Group Treatment Protocol (EMDR-IGTP) Applied to Caregivers of Patients With Dementia

**DOI:** 10.3389/fpsyg.2018.00967

**Published:** 2018-06-15

**Authors:** Serena Passoni, Teresa Curinga, Alessio Toraldo, Manuela Berlingeri, Isabel Fernandez, Gabriella Bottini

**Affiliations:** ^1^Cognitive Neuropsychology Center, ASST Grande Ospedale Metropolitano Niguarda, Milan, Italy; ^2^EMDR Italy Association, Varedo, Italy; ^3^Department of Brain and Behavioral Sciences, University of Pavia, Pavia, Italy; ^4^Milan Center for Neuroscience, Milan, Italy; ^5^Department of Humanistic Study, University of Urbino “Carlo Bo”, Urbino, Italy; ^6^Center for Developmental Neuropsychology, Pesaro, Italy

**Keywords:** dementia, caregivers, EMDR Integrative Group Treatment Protocol, anxiety, depression, burden

## Abstract

Caregivers of patients with dementia experience high levels of stress and burden, with effects comparable to those of a traumatic event. Eye Movement Desensitization and Reprocessing (EMDR) appear to be effective in recovering post-traumatic stress disorder (PTSD). We aimed at investigating the effectiveness of the Eye Movement Desensitization and Reprocessing Integrative Group Treatment Protocol (EMDR-IGTP) on the “caregiver syndrome”. Forty-four primary caregivers entered the study. They were randomly assigned to either the “immediate” branch, who received the treatment soon after recruitment, or to the “delayed” branch, who received it two months after recruitment. The treatment consisted of eight group sessions (one per week) spanning over two months. Emotional distress was measured before the treatment, immediately after the end of it, and two months later (follow-up), by means of several clinical scales (Impact of Event Scale-Revised, IES-R; Caregiver Needs Assessment, CNA; Caregiver Burden Inventory, CBI; Anxiety and Depression Scale-Reduced Form, AD-R). The “immediate” branch improved significantly more than the “delayed” (control) branch on The Impact of Event Scale-Revised, the Anxiety, and the Depression scales; however, after treatment such an improvement was maintained only in the first scale. The “delayed” branch took less advantage of the treatment, showing significant reduction only on the Depression scale, an effect which disappeared at follow-up. These preliminary results show for the first time that EMDR-IGTP reduces stress-related symptoms, anxiety, and depression in caregivers of patients with dementia. Interestingly, caregivers who were inserted in a waiting list after recruitment showed smaller treatment effects. Larger samples are needed to better interpret such differential clinical profiles.

## Introduction

Dementia is a degenerative disease with a major impact on the whole family of the patient (Beinart et al., 2012), especially on primary caregivers. Prolonged care of patients with dementia is associated with somatic and psychological symptoms that characterize the “caregiver syndrome” (Gaugler et al., 2005). This syndrome together with wrong coping strategies may culminate in high risk of developing affective disorders, with high levels of stress, anxiety, depression (Cuijpers, 2005; Gaugler et al., 2005), and burden (Vitaliano et al., 2003; Passoni et al., 2010). The Behavioural and Psychological Symptoms of Dementia (BPSD), as well as the progressive disability in performing basic activities of daily life, have a negative impact on the immune system of the caregiver (Kiecolt-Glaser et al., 1991), inducing a decline in physical health with the rise of emotional and affective disorders (Dunkin and Anderson-Hanley, 1998; Burns, 2000).

Caregivers of patients with dementia experience such symptoms soon after diagnosis. Several studies show that caregivers have higher levels of psychiatric and physical morbidity and use psychotropic drugs more frequently than other family members who are not directly involved in the assistance (Dunkin and Anderson-Hanley, 1998; Burns, 2000). In a nutshell, the caregiver becomes a “secondary victim” of the disease, a problem that in turn reduces his/her competence in caring.

For all these reasons, being involved in the assistance of a patient with dementia can well be considered as a traumatic event. Worse still, taking care of a patient with dementia exposes the caregiver to multiple traumatic events – the daily contact with the patient exposes him/her to repeated and prolonged stress triggers, similar to the acute trauma of the initial diagnosis in their effects (Freedman et al., 1999; Jarero and Uribe, 2011, 2012). This multi-traumatic sequence makes caregivers more likely to show symptoms of post-traumatic stress disorder (PTSD) than individuals who experienced a single stressful event (McFarlane, 1989; Uddo et al., 1996).

Canonical strategies for reducing caregivers’ distress include pharmacotherapy and psychosocial interventions such as psychotherapy (cognitive-behavioral in focus, e.g., Passoni et al., 2014) and psycho-educational programs (Pinquart and Sorensen, 2006; Cooper et al., 2007; Gallagher-Thompson and Coon, 2007; Elvish et al., 2012). These interventions mainly focus on practical issues concerning disease managing (Gallagher-Thompson et al., 2010; Elvish et al., 2012) and neglect the traumatic event experienced by the caregiver. We wished to take into account this aspect by treating caregivers with the Eye Movement Desensitization and Reprocessing (EMDR) technique.

EMDR was developed by Shapiro (2001) and Shapiro and Maxfield (2002) and is often used to treat PTSD. The World Health Organization [WHO] (2013) and several international guidelines (e.g., Cochrane Review) recommend EMDR for treating PTSD in children, adolescents and adults (Bisson and Andrew, 2007). The alternation of eye movement or tactile/auditory stimulation represents the core of this therapy, which is held to favor the elaboration of the trauma on which patients are focusing.

Because of its effectiveness with PTSD, the use of EMDR has been extended to sexual and physical abuse, bereavement, or abortion, with apparently reduction of the emotional distress. As a consequence of the flood caused by the Pauline Hurricane in Mexico (1997), a huge demand of urgent psychotherapeutic intervention occurred that overwhelmed the mental health services. Psychotherapists of the Mexican Association for Mental Health Support in Crisis (AMAMECRISIS; Jarero et al., 2008; Jarero and Artigas, 2009) decided to administer EMDR to large groups of children, thus developing the EMDR Integrative Group Treatment Protocol (EMDR-IGTP) for early intervention.

This protocol, originally designed for children (Artigas et al., 2014) was later adapted for adults (Jarero and Artigas, 2014) and used with appropriate modifications in different circumstances around the world (Maxfield, 2008; Jarero and Artigas, 2012). EMDR appears to be effective when compared to other group treatments in terms of time, resources and outcome (Adúriz et al., 2009).

Two broad categories of application contexts are considered. The first concerns large groups of people who experienced the same critical event, such as natural and man-made disasters (Jarero et al., 2006, 2008; Errebo et al., 2008; Jarero and Uribe, 2012) or traumatic events with an impact on small communities (suicide of a boy, murders, etc.). The second concerns people experiencing the same type of trauma, although in separate critical events (e.g., rescuers, parents of disabled children, patients with cancer, etc.; Jarero et al., 2014).

A recent pilot study showed that EMDR-IGTP was effective in 24 women with cancer diagnosed with PTSD (Jarero et al., 2014).

However, overall, evidence on the effectiveness of EMDR-IGTP is still scanty.

To our knowledge, EMDR has never been used in caregivers of patients with dementia, and this would be a suitable population given the frequency of PTSD symptoms within it. Hence, the aim of the present work was to test whether EMDR-IGTP is effective in reducing post-traumatic and emotional symptoms (anxiety, burden, depression, needs related to care) in dementia patients’ caregivers.

## Materials and Methods

### Participants

Caregivers of patients with dementia were recruited at the Memory Clinic of the Cognitive Neuropsychology Centre of the Niguarda Hospital, in Milan. Potential caregivers were informed on the opportunity to attend the study by the neurologist during the clinical evaluation of the patient. A caregiver entered the trial only if s/he met the inclusion/exclusion criteria listed below and if s/he gave written informed consent to the participation after having been informed about the objectives of the study. If a caregiver gave informed consent, a set of further, relevant clinical variables regarding the patient (MMSE, ADL, IADL), were collected by the physician during the neurological evaluation.

The study was approved by the Local Ethics Committee of the Niguarda Hospital (September 18th, 2015, approval number 443-092015) and was conducted following the principles for standards of Good Clinical Practice.

#### Inclusion Criteria

- Being a caregiver of a patient with a diagnosis of dementia on grounds of the DSM-IV (American Psychiatric Association [APA], 1994) criteria.
- Being the *primary* caregiver (the one most involved in the care in terms of time).
- Being a relative of the patient.
- Having assisted the patient for at least six consecutive months, at home (in this way we could guarantee safer AD diagnoses and stability of the stressful caregiver–patient relationship).
- Showing evidence of one or more traumatic events causing trauma related symptoms (IES-R > 0, and Subjective Units of Distress, SUD > 5).
- Being fluent in Italian and with at least three years of education.

#### Exclusion Criteria

- Evidence of severe psychiatric disorders.

### Study Design

**FIGURE 1:**
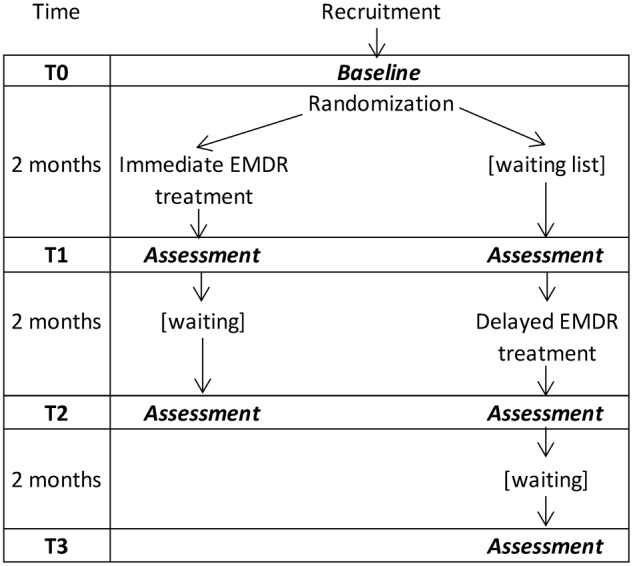
Study design.

The study was monocentric, single-blind, and had two parallel branches (Schulz et al., 2010), thus conforming to an Individually Randomized Group Treatment Trial. The clinical effect of the EMDR-IGTP treatment in each branch was assessed at three time points (T0, T1, and T2) plus another time point, T3, for the second branch. Time points were two months apart (see **Figure 1**). Examiners who administered the clinical tests at each time point were blind to the branch of the evaluated caregiver.

In the first branch (“Immediate” EMDR-IGTP condition), therapy was administered between T0 and T1; in the second branch (“Delayed” EMDR-IGTP, or “Waiting List” condition) therapy was administered between T1 and T2. This branch did not undergo any intervention between T0 and T1, so it served as a control condition to be compared to the “Immediate” one. The direct effect of the EMDR treatment was thus quantified in terms of the differential improvement between T0 and T1 in the two branches.

After the initial assessment (T0) caregivers were randomly assigned to one of the two branches. Randomization was carried out by assigning one caregiver to one branch and the next to the other branch, on grounds of mere arrival order. This randomization technique (with the source of randomness being arrival order) was necessary in order to closely synchronize the two branches in their successive T1 and T2 assessments, thus matching every variable related to time-of-the-year between conditions (indeed, seasonal changes, like depression level, might be a source of confusion, Postolache et al., 1998).

The date when treatment sessions started was determined by practical constraints (e.g., the need to avoid interruptions because of holiday periods) and/or by the number of applicants assigned to the immediate condition reaching 10, that is, the maximum number of caregivers that were allowed to join in a single treatment group. Note that the term “group” will henceforth exclusively refer to a set of caregivers who attended the same treatment sessions. At the end of the study, six groups were formed, three per branch, with 7, 5, and 10 participants (Immediate branch) and 8, 4, and 10 participants (Delayed branch).

### Measures

#### Caregiver Variables

The following questionnaires/tests were administered to the caregiver at each time point.

• A data form to collect clinical and socio-demographic features (age, gender, educational level, patient–caregiver kinship, caring time measure i.e., number of weekly and/or daily hours, duration of the caregiving role in months).
• *Visual Analog Scale: VAS*, a paper-and-pencil version of the Likert scale. Caregivers were asked to point to a graduated horizontal line (a 0–10 ruler) to rate their subjective perception of (i) the quality of the premorbid relationship with the patient, (ii) the severity of the patient’s disease, and (iii) the relative speed of the evolution of the disease.
• *Impact of Event Scale-Revised: IES-R* (Horowitz et al., 1979; Weiss and Marmar, 1997). This 22-item self-report is useful for assessing subjective distress caused by traumatic events. Patients are asked to identify a specific stressful event and indicate how much they were distressed by it during the past 7 days. Items are rated on a 5-point Likert scale ranging from 0 (“not at all”) to 4 (“extremely”). IES-R yields a 0–88 total score and specific subscale scores (Intrusion, Avoidance, Hyperarousal). IES-R is the most widespread self-administered measure of PTSD symptoms.
• *Caregiver Burden Inventory: CBI* (Novak and Guest, 1989). This scale quantifies burden and contains five different sections: Time-dependence Burden (items 1–5), Developmental Burden (items 6–10), Physical Burden (items 11–14), Social Burden (items 15–19), and Emotional Burden (items 20–24). CBI’s 24 items yield an overall 0–96 score.
• *Anxiety and Depression Scale-Reduced Form: AD-R* (Moroni et al., 2006). This tool was validated for patients in rehabilitation setting and consists of 25 items, 15 of which (range: 0–15) constitute the Depression Questionnaire Reduced Form (QD-R; Vidotto et al., 2010), and 10 of which (range: 10–40) constitute the State Anxiety Inventory – Reduced Form (STAI-X3; Spielberger et al., 1970; Vidotto and Bertolotti, 1991).
• *Caregiver Need Assessment: CNA* (Moroni et al., 2008) was used to assess the caregivers’ needs related to care. This questionnaire consist of 17 items with 0–3 Likert responses (overall score: 0–51 the higher, the higher the level of need) and includes two subscales (which proved to be internally consistent) labeled “Needs of emotional and social support”, CNA-1 (Cronbach α = 0.765) and “Needs of information and communication”, CNA-2 (Cronbach α = 0.742).

#### Patient Variables

• *Mini Mental State Examination: MMSE* (Folstein et al., 1975; Measso et al., 1993), a widespread screening test, was administered to assess the patient’s state of dementia; it samples various cognitive functions such as memory and orientation, and has a 0–30 range. Scores were adjusted for age and education (MMSE corr, Measso et al., 1993).
• *Instrumental Activities of Daily Living: IADL* (Lawton and Brody, 1969) with scores ranging 0–6, and *Activities of Daily Living: ADL* (Katz et al., 1963) with scores ranging 0–8, were used to estimate the patient’s degree of autonomy in basic daily living activities and his/her ability to take care of his/her own person.

### EMDR-IGTP Intervention

Two psychotherapists held the EMDR-IGTP sessions, an EMDR practitioner and an EMDR trainer.

All caregivers received eight group sessions of 120 min each, covering a 2-month period. The main protocol included the following steps.

(a) A first session delivered information as to the main characteristics of dementia and as to how to manage the behavioral and psychological symptoms of the disease. Caregivers were provided with suggestions concerning healthy behaviors for stress management and physical / psychosocial activities.
(b) A second session provided an assessment of dysfunctional cognitions in the context of the traumatic event of taking care of a person with dementia. In this session caregivers were trained by means of imagery exercises and stabilization techniques, such as “the safe place” (Shapiro, 2001), which can be practiced also at home as a strategy to reduce distress.
(c) The following sessions were dedicated to the re-processing of traumatic events through the EMDR-IGTP. This protocol combines the eight phases of the EMDR Individual Therapy treatment (Shapiro, 2001) in a group therapy model and an art therapy format. In EMDR-IGTP sessions, each caregiver is asked to focus upon the traumatic memory or highly stressful recollections related to the relative’s disease. There is no verbalization of these contents: the caregiver is instructed to produce some drawings on a paper sheet that are related to the painful memories s/he is experiencing (after every image drawn, the level of distress is monitored by means of a “subjective units of discomfort” – SUD – rating scale). S/he is then required to focus upon the just-produced drawings, while simultaneously self-administering a form of bilateral self-stimulation known as the “butterfly hug” – with each self-stimulation lasting for approximately 45 s (Group Butterfly Hug Protocol, Artigas and Jarero, 2014). Towards the end of the group session, caregivers are asked whether they experienced some positive memories or feelings during the butterfly hug, and if so, they are asked to produce drawings relative to these, in order to close the session with a self-stimulation related to positive contents.

### Statistical Analyses

The analyses were run in the R-studio (version: 1.0.143) environment using *ad hoc* created routines^1^ based on the standard libraries available online. We started by exploring the relationship between clinical and socio-demographical variables by means of non-parametric Spearman’s rank correlation test on the basis of specific *a priori* hypotheses.

As a second step, the clinical variables (namely IES-R, CBI, CNA, and Depression and Anxiety scales) were normalized according to the following formula:

$$\begin{array}{c} \mathrm{Normalized}\;\mathrm{score}\;x\;\;=\;\;(x{-min}_{x})/({\mathrm{MAX}}_{x}{-min}_{x}) \end{array}$$

This normalization was carried out in order to make all the clinical variables fully comparable with one another (bounds all became 0–1).

The normalized scores were then entered as dependent variables into a series of generalized linear mixed model with random intercept (grouped by subject) and with time (T0 vs. T1 vs. T2) and branch (Immediate vs. Delayed) as fixed effect predictors. Moreover, Intra-Class Correlations (ICC) were computed to ascertain whether the administration of treatment on separate groups produced critical violations of the assumption of statistical independence among observations (Searle, 1971; Thomas and Hultquist, 1978; Donner, 1979). **Table 1** reports the ICC values, which clearly indicate that the adoption of separate groups did not create any cluster of data. Hence, the random intercept was modeled only by subjects. In particular, these analyses were run using the lme4 package:

**Table 1:** ICC indices and 95% confidence intervals for the six “groups” of caregivers.

	ICC index	CI lower bound	CI upper bound
**EMDR-efficacy**			
IES-R	–0.025	–0.045	0.110
CNA	0.065	–0.008	0.411
CBI	0.078	–0.002	0.442
Anxiety	–0.041	–0.051	0.031
Depression	0.049	–0.014	0.372
**FU-analyses**			
IES-R	–0.006	–0.062	0.295
CNA	–0.024	–0.070	0.235
CBI	0.0362	–0.045	0.406
Anxiety	–0.064	–0.085	0.076
Depression	–0.009	–0.064	0.283

*FU, Follow-Up.*

$$\begin{array}{c} MODEL\;X\;=\;\mathrm{lmer}(\mathrm{NORMALIZED}\;\mathrm{SCORE}\;X\sim \mathrm{BRANCH}*\mathrm{TIME}+(1|SUBJECT),\mathrm{data}\;=\;\mathrm{mydata}) \end{array}$$

The fixed effect marginal means were then extracted to plot the first and the second level effects; moreover, if significant, the BRANCH-by-TIME interaction effect was further explored by means of pairwise comparisons while adopting a FDR correction for multiple comparisons. In the case of the CNA, CBI, IES-R variables, if the BRANCH-by-TIME interaction effect was significant in the overall score, we further explored the same interaction within each subscale.

It is worth noting here that we were particularly interested in the BRANCH-by-TIME interaction as, according to our study design, that should genuinely reflect the effectiveness of the EMDR-IGTP treatment.

Finally, in order to explicitly evaluate the persistence of the EMDR-IGTP in the follow-up phase, we isolated the data collected at the end of the treatment in the two branches of caregivers (namely in the Immediate and Delayed branches) and the data collected after 2 months (i.e., the specific follow-up phase for each branch) and designed a new series of generalized linear mixed model with random intercept (grouped by subject) with time (post-treatment vs. follow up) and branch (immediate vs. delayed) as fixed effect predictors. These analyses were run using the lme4 package too. For all the *post hoc* comparisons, an FDR correction for multiple comparison was applied (R package “phia”; De Rosario-Martinez, 2013).

## Results

### Socio-Demographical and Clinical Description of the Two Branches

We initially recruited 44 caregivers, 22 per branch; 11 of them dropped out of the study during the EMDR-IGTP intervention, eight from the Delayed condition, three from the Immediate condition (the difference between the two drop-out rates was not significant, χ^2^ = 3.03, *p* = 0.082). Apart from drop-outs, there were no missing data: all caregivers yielded a complete dataset in all sessions in which they participated. Given this lack of evidence of differential drop-out rates, we applied an *intention-to-treat* approach (Gupta, 2011), thus including dropped-out caregivers in the analyses (incidentally, this is the default choice of the mixed linear model approach). To test the appropriateness of such a choice, we also ran control *per-protocol* analyses (excluding drop-outs); given that per protocol-analyses yielded very similar results to those obtained by the intention-to-treat approach, we reported only the latter as they are based on slightly larger sample sizes.

#### Socio-Demographic Characteristics

Among the 44 caregivers, 34 were females and were most often the spouses (*N* = 30) of the patient. They had a mean age of 66.07 years (*SD* = 11.32), and an education level of 11.04 years (*SD* = 4.09).

Caregivers have been taking care of the patient for an average of 32.68 months (*SD* = 22.84). Thirty-two caregivers were living with the patient and most of them were involved in the care almost every day (mean = 6.2 days a week, *SD* = 1.74).

Half the caregivers did not receive help of any kind (*N* = 22), the others could count on some help from a third party (see **Table 2** for details).

**Table 2:** Socio-demographic characteristics of caregivers.

	Immediate branch	Delayed branch
	Mean (*SD*)	Mean (*SD*)
Age (years)	64.9 (± 13.04)	67.22 (± 9.48)
Education (years)	12.45 (± 3.83)	9.63 (± 3.93)^∗^
Caring time (number of days per week)	6.04 (± 1.86)	6.36 (± 1.64)
Caring duration (months since diagnosis)	34.41 (± 27.55)	30.95 (± 17.4)
	**#**	**#**
Sex of the caregivers		
∙ Female	16	18
∙ Male	6	4
Caregivers’ Kinship status • Spouse • Son/daughter • Brother/sister	12 9 1	18 4 0
Caregivers’ living status • With the patient • Elsewhere	14 8	18 4
Type of help received • No help • By a relative • By a formal carer • By a relative and a formal carer • By a friend	8 9 3 1 1	14 4 3 1 0

*^∗^significant between-branches differences (*p* < 0.05). Between-branches comparisons were run using a Wilcoxon Mann–Whitney U-test as implemented in R.*

**Table 2** also reports demographics separately for the two conditions.

Caregivers included in our sample had generally homogeneous socio-demographic characteristics. The two branches did not differ on demographic variables, with the exception of educational level which was higher in the Immediate than in the Delayed branch (*t*(42) = 2.405, *p* = 0.02).

#### Correlations Between Clinical Variables at the Enrolment Phase

The relationships between the different clinical variables were explored on the scores obtained by the entire sample at enrolment (T0). We found a significant negative Spearman correlation between the level of burden of the caregiver (CBI) and the level of autonomy by the patient in daily activities (IADL; ρ = -0.34; S = 14390, *p*-value = 0.026) which suggests that the lower the patients’ residual abilities of daily living, the higher the level of caregivers’ burden. This correlation was particularly pronounced for the “Time” subscale (ρ = -0.41; S = 15074, *p*-value = 0.008).

Similarly, we found a significant negative correlation between the overall level of caregiver’s burden and the perceived quality of the premorbid patient-caregiver relationship (ρ = -0.34; S = 19005, *p*-value = 0.024): the lower the quality of the relationship, the higher the level of burden. This correlation was particularly strong for the “Social” (ρ = -0.35; S = 19177, *p*-value = 0.019) and the “Physical” (ρ = -0.42; S = 20147, *p*-value = 0.004) subscales.

### Effect of the EMDR-IGTP Intervention

As described in Section “Materials and Methods”, we ran a series of linear mixed models with by-subject random intercept to test the BRANCH-by-TIME interaction effect.

In what follows, we report the main effect and the interaction effect for the overall scores of our clinical variables.

**FIGURE 2:**
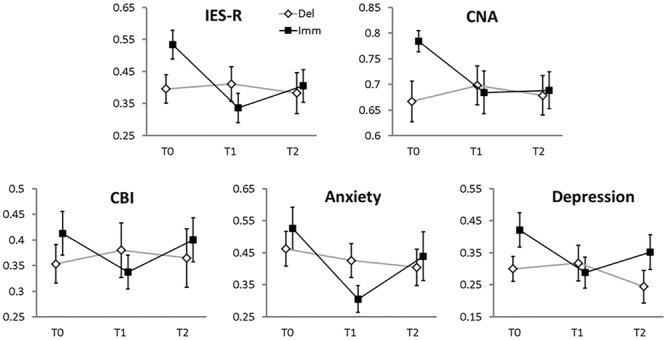
Mean values (error bars: standard errors) of the main clinical variables (standardized to the 0–1 scale, 0 = minimum 1 = maximum score, reported on the *y*-axes). The *x*-axes report the time of assessment: T0, enrolment phase; T1, 2 months later (i.e., the end of the therapy for the Immediate branch, the end of the waiting-list period for the Delayed branch); T2, another 2 months later (the end of the therapy for the Delayed branch; follow-up visit for the Immediate branch). Filled squares, Immediate branch; open diamonds, Delayed branch.

(a) **IES-R:** we could not find a main effect of BRANCH (χ^2^ = 1.4, *df* = 1, *p*-value = 0.23), but there was a significant main effect of TIME (χ^2^ = 12.03, *df* = 2, *p*-value = 0.002) and a significant BRANCH-by-TIME interaction effect (χ^2^ = 8.72, *df* = 2, *p*-value = 0.01). As shown in **Figure 2**, the interaction effect was due to a significant decrement of the IES-R score between T0 and T1 (χ^2^ = 18.61, *df* = 1, *p*-value < 0.001) and between T0 and T2 (χ^2^ = 7.22, *df* = 1 *p*-value = 0.02) in the Immediate condition only (FDR-corrected comparisons).

(b) **CNA:** we could not find a main effect of BRANCH (χ^2^ = 2.66, *df* = 1, *p*-value = 0.1); neither did we find a significant main effect of TIME (χ^2^ = 4.05, *df* = 2, *p*-value = 0.13), or a significant BRANCH-by-TIME interaction effect (χ^2^ = 4.29, *df* = 2, *p*-value = 0.11).
(c) **CBI:** in this analysis no significant main effect of BRANCH emerged (χ^2^ = 0.5, *df* = 1, *p*-value = 0.47); neither a significant main effect of TIME (χ^2^ = 2.22, *df* = 2, *p*-value = 0.33), nor a significant BRANCH-by-TIME interaction effect (χ^2^ = 5.06, *df* = 2, *p*-value = 0.08) could be found.
(d) **Anxiety:** albeit there was no significant main effect of BRANCH (χ^2^ = 0, *df* = 1, *p*-value = 0.99), a significant main effect of TIME emerged (χ^2^ = 8.26, *df* = 2, *p*-value = 0.01). The BRANCH-by-TIME interaction effect was not significant (χ^2^ = 4.81, *df* = 2, *p*-value = 0.09).
(e) **Depression:** we could not find a main effect of BRANCH (χ^2^ = 1.8, *df* = 1, *p*-value = 0.18), but there was a significant main effect of TIME (χ^2^ = 7.36, *df* = 2, *p*-value = 0.02) and a significant BRANCH-by-TIME interaction effect (χ^2^ = 11.9, *df* = 2, *p*-value = 0.002). As shown in **Figure 2**, the interaction effect was due to a significant decrement of the Depression score between T0 and T1 (χ^2^ = 13.43, *df* = 1, *p*-value = 0.001) in the Immediate condition, on the one hand, and between T1 and T2 (χ^2^ = 5.55, *df* = 1 *p*-value = 0.05) in the Delayed condition, on the other hand (FDR-corrected comparisons).

As described in Section “Materials and Methods”, we further explored the BRANCH-by-TIME interaction effect in the subscales of the IES-R measure. In particular, for the “Avoidance” subscale a significant BRANCH-by-TIME interaction effect emerged (χ^2^ = 6.4, *df* = 2, *p*-value = 0.04) in the absence of significant main effects (BRANCH: χ^2^ = 2.80, *df* = 1, *p*-value = 0.09; TIME: χ^2^ = 3.04, *df* = 2, *p*-value = 0.21). The pairwise FDR-corrected comparisons showed that the BRANCH-by-TIME interaction effect in the “Avoidance” subscale was due to a significant difference between T0 and T1 (χ^2^ = 7.36, *df* = 1 *p*-value = 0.04) and T0 and T2 (χ^2^ = 5.9, *df* = 1 *p*-value = 0.04) in the Immediate condition only. In the “Intrusion” subscale we found a significant main effect of TIME (χ^2^ = 15.32, *df* = 2, *p*-value < 0.001) and a significant BRANCH-by-TIME interaction effect (χ^2^ = 6.36, *df* = 2, *p*-value = 0.04). The pairwise FDR-corrected comparisons showed that the BRANCH-by-TIME interaction effect in the “Intrusion” subscale was due to a significant difference between T0 and T1 (χ^2^ = 19.71, *df* = 1 *p*-value < 0.001) and T0 and T2 (χ^2^ = 6.15, *df* = 1 *p*-value = 0.04) in the Immediate condition only. A similar pattern of results emerged also for the “Hyperarousal” subscale: a main effect of TIME (χ^2^ = 15.33, *df* = 2, *p*-value < 0.001) and a significant BRANCH-by-TIME interaction effect (χ^2^ = 13.09, *df* = 2, *p*-value = 0.001) emerged. The pairwise FDR-corrected comparisons showed that the BRANCH-by-TIME interaction effect in the “Hyperarousal” subscale was due to a significant difference between T0 and T1 (χ^2^ = 27.87, *df* = 1 *p*-value < 0.001) and T0 and T2 (χ^2^ = 7.9, *df* = 1 *p*-value = 0.01) and T1 and T2 (χ^2^ = 5.4, *df* = 1 *p*-value = 0.04) in the Immediate condition only.

### Stability of the EMDR-IGTP Intervention at the Follow-Up Phase

**FIGURE 3:**
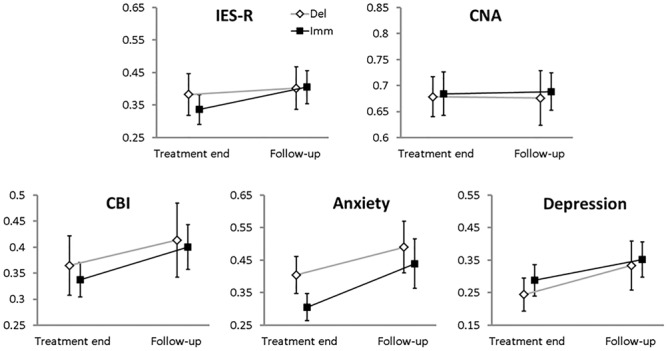
Means (error bars: standard errors) for the 0–1 standardized main clinical variables collected at the end of the therapy and at the follow-up phase for each branch. Filled squares, Immediate branch; open diamonds, Delayed branch.

As a final step, we evaluated the persistence of the EMDR-IGTP effect in the follow-up phase, i.e., two months after the last treatment session (**Figure 3**). Also, in this case ICC were far from significance (they ranged from -0.065 to 0.036).

(a) **IES-R:** no effect was significant (BRANCH, χ2 = 0.075, *df* = 1, *p*-value = 0.78; TIME, χ2 = 3.3, *df* = 1, *p*-value = 0.07; BRANCH-by-TIME, χ2 = 0.09, *df* = 1, *p*-value = 0.76).
(b) **CNA:** we could find neither a main effect of BRANCH (χ^2^ = 0.02, *df* = 1, *p*-value = 0.89), nor of TIME (χ^2^ = 0.04, *df* = 1, *p*-value = 0.84), nor a BRANCH-by-TIME interaction effect (χ^2^ = 0.02, *df* = 1, *p*-value = 0.87).
(c) **CBI:** we could not find a main effect of BRANCH (group, χ^2^ = 0.07, *df* = 1, *p*-value = 0.78) but a significant main effect of TIME emerged (χ^2^ = 8.01, *df* = 1, *p*-value = 0.004); the BRANCH-by-TIME interaction effect was not significant (χ^2^ = 0.007, *df* = 1, *p*-value = 0.93).
(d) **Anxiety:** no main effect of BRANCH (χ^2^ = 0.82, *df* = 1, *p*-value = 0.36) or BRANCH-by-TIME interaction effect emerged (χ^2^ = 0.23, *df* = 1, *p*-value = 0.63); however a significant effect of TIME (χ^2^ = 9.93, *df* = 1, *p*-value = 0.001) was found.
(e) **Depression:** we did not find a main effect of BRANCH (χ^2^ = 0.29, *df* = 1, *p*-value = 0.58), but a significant main effect of TIME (χ^2^ = 7.14, *df* = 1, *p*-value = 0.007) emerged. The interaction BRANCH-by-TIME was not significant (χ^2^ = 0.24, *df* = 1, *p*-value = 0.62).

## Discussion

The purpose of the present study was to learn whether EMDR-IGTP could be proved effective in the treatment of the symptoms of emotional distress shown by primary caregivers of patients with dementia. We administered EMDR-IGTP to two randomized branches of caregivers, the former starting the treatment immediately after consent (Immediate), the latter inserted on a 2-month waiting list (Delayed).

We found two expected, negative correlations in the initial, enrolment phase: namely, the level of burden of the caregiver was inversely proportional to the level of autonomy of the patient in daily activities (IADL); moreover, caregivers describing a poor quality of the premorbid relationship with the patient had higher levels of social and physical burden.

The evaluation of the effectiveness of EMDR-IGTP in reducing post-traumatic distress symptoms in caregivers – the primary purpose of the present work – could be carried out by comparing the evolution of clinical scores in the Immediate vs. Delayed conditions. Indeed, between T0 (the time of baseline assessment) and T1 (2 months later) caregivers of the Immediate branch received the treatment, while those of the Delayed branch did not receive any treatment and remained in the waiting list. As expected, EMDR-IGTP treatment significantly reduced the level of subjective distress related to the traumatic event in the Immediate condition, while no detectable change was observed in the Delayed condition. This pattern was confirmed in all of the three IES-R subscales: treated caregivers showed a reduction of Intrusion, Avoidance, and Hyperarousal symptoms. Caregivers of the Immediate branch also showed a reduction in anxiety and an improvement of mood, with a decrease of the levels of depression. The reduction of distress (IES-R) was maintained after another 2 months (i.e., 2 months after interruption of the treatment), while anxiety, depression and burden (CBI) showed an increase in the same period.

The IES-R results mirror those of other studies focusing on EMDR and EMDR-IGTP on other populations, like patients with physical diseases (cancer or multiple sclerosis: Capezzani et al., 2013; Jarero et al., 2014; Carletto et al., 2016), albeit in the same studies depression and anxiety kept stable at follow-up.

In the Delayed condition, in which the caregivers received the EMDR-IGTP treatment later (between two and four months after initial enrolment and screening), a significant treatment effect was observed only on the depression scale. As for the follow-up, two months after the end of the treatment, the effects were virtually identical to those recorded from the Immediate branch, that is, a worsening of the anxiety and depression symptoms as well as an increase of burden (CBI).

In our experimental design caregivers of both branches received information on the EMDR treatment at the time of initial assessment; thus all caregivers probably developed positive expectations about the treatment – we have no reason to believe that the degree of such initial expectations was any different in the two branches, since caregivers were randomized into one of them after that initial assessment. We believe one explanation of this complex results profile is the following. During the 2 months in which caregivers of the Delayed branch had to wait before treatment began, a significant number of further stressful events related to caregiving occurred, against which they had no defense (yet). Indeed, it is well known that the severity of the patient’s (often progressive) inability to perform basic activities of daily life, as well as his/her behavioral and psychological symptoms (BPSD) contribute to maintain high levels of stress, associated to emotional and affective disorders and burden (e.g., Dunkin and Anderson-Hanley, 1998; Burns, 2000; Vitaliano et al., 2003; Cuijpers, 2005; Gaugler et al., 2005; Passoni et al., 2010). Two more months without tools to stem the negative effects of the sequence of stressful events might have made the caregivers less responsive to the EMDR-IGTP treatment.

Finally, it is worth noting that although other studies explored the effectiveness of psychological treatments on caregivers, they typically compared the Immediate to the Delayed (“waiting list”) condition, without exploring the effects of the therapy in the Delayed condition (Gallagher-Thompson et al., 2000; Akkerman and Ostwald, 2004). Our study also explored such effects.

Albeit preliminary, the present study is (to our knowledge) the first description of the effects of treatment timing. Further studies are needed to better understand the behavioral components characterizing caregivers in this different time frame.

Wrapping up, the issues raised in this discussion are relevant in the clinical setting: our study suggests that an *early* intervention is the best response to the difficulties experienced by caregivers of patients with dementia. Indeed, such an intervention would enable them to better cope with the unavoidable sequence of stressful events yielded by their relatives’ condition. Without an early intervention, the steep progression of the patients’ disease might worsen the emotional condition of caregivers so much as to make them less responsive to treatment.

### Limits

The present results, albeit intriguing, need further investigation. In particular, we plan to extend the sample and to collect data from later follow-ups: indeed the suggestion that delaying the treatment might produce a loss of the positive treatment effects needs further scrutiny. Another issue is the stabilization of the positive effects 2 months after the end of the treatment, which regarded distress symptoms but not depression and anxiety, which tended to increase again. Perhaps 2 months of treatment were enough for producing sizeable positive effects, but not enough for stabilizing them and/or generalizing them to all problematic sectors of the caregiver’s mental status. Whether or not longer treatment periods, possibly covering various phases of the patients’ degenerative disease, produce more stable effects is an empirical question.

## Conclusion

The present study evaluates for the first time the efficacy of the EMDR-IGTP treatment in caregivers of patients with dementia.

Three of the five tested measures (Impact of Event Scale-Revised, Anxiety, and Depression) witnessed a clear and multifaceted improvement related to a therapy that lasted only 2 months. These preliminary data suggest that EMDR-IGTP might be considered as a valid tool to reduce distress symptoms in caregivers of patients with dementia.

## Author Contributions

SP and IF were responsible for the conception of the study. SP and AT designed the study. SP and TC conducted the study. MB and AT were responsible for data collection and statistical analyses. SP and MB wrote the article, which was critically revised by AT, TC, IF, and GB. All authors read and approved the final version of the manuscript, and guarantee the accuracy and integrity of this work in all its aspects.

## Conflict of Interest Statement

IF is the president of the EMDR Europe and Italian Associations. The remaining authors declare that the research was conducted in the absence of any commercial or financial relationships that could be construed as a potential conflict of interest.

## Footnotes

1 The R script can be obtained by emailing MB.
